# Impairment of Protein Trafficking upon Overexpression and Mutation of Optineurin

**DOI:** 10.1371/journal.pone.0011547

**Published:** 2010-07-12

**Authors:** BumChan Park, Hongyu Ying, Xiang Shen, Jeong-Seok Park, Ye Qiu, Rajalekshmy Shyam, Beatrice Y. J. T. Yue

**Affiliations:** Department of Ophthalmology and Visual Sciences, University of Illinois at Chicago College of Medicine, Chicago, Illinois, United States of America; Johns Hopkins University, United States of America

## Abstract

**Background:**

Glaucoma is a major blinding disease characterized by progressive loss of retinal ganglion cells (RGCs) and axons. Optineurin is one of the candidate genes identified so far. A mutation of Glu^50^ to Lys (E50K) has been reported to be associated with a more progressive and severe disease. Optineurin, known to interact with Rab8, myosin VI and transferrin receptor (TfR), was speculated to have a role in protein trafficking. Here we determined whether, and how optineurin overexpression and E50K mutation affect the internalization of transferrin (Tf), widely used as a marker for receptor-mediated endocytosis.

**Methodology/Principal Findings:**

Human retinal pigment epithelial (RPE) and rat RGC5 cells transfected to overexpress wild type optineurin were incubated with Texas Red-Tf to evaluate Tf uptake. Granular structures or dots referred to as foci formed in perinuclear regions after transfection. An impairment of the Tf uptake was in addition observed in transfected cells. Compared to overexpression of the wild type, E50K mutation yielded an increased foci formation and a more pronounced defect in Tf uptake. Co-transfection with TfR, but not Rab8 or myosin VI, construct rescued the optineurin inhibitory effect, suggesting that TfR was the factor involved in the trafficking phenotype. Forced expression of both wild type and E50K optineurin rendered TfR to colocalize with the foci. Surface biotinylation experiments showed that the surface level of TfR was also reduced, leading presumably to an impeded Tf uptake. A non-consequential Leu^157^ to Ala (L157A) mutation that displayed much reduced foci formation and TfR binding had normal TfR distribution, normal surface TfR level and normal Tf internalization.

**Conclusions/Significance:**

The present study demonstrates that overexpression of wild type optineurin results in impairment of the Tf uptake in RPE and RGC5 cells. The phenotype is related to the optineurin interaction with TfR. Our results further indicate that E50K induces more dramatic effects than the wild type optineurin, and is thus a gain-of-function mutation. The defective protein trafficking may be one of the underlying bases why glaucoma pathology develops in patients with E50K mutation.

## Introduction

Glaucoma, a major blinding disease worldwide [1], is characterized by progressive loss of retinal ganglion cells (RGCs) and axons, as well as cupping of the optic nerve head. The most common form of this disease, primary open angle glaucoma (POAG) is frequently associated with elevated intraocular pressure (IOP) [2].

Recent genetic studies have demonstrated that POAG is highly heterogeneous, caused by several susceptibility genes and perhaps also environmental factors [1]–[5]. To date, three candidate genes, myocilin, optineurin and WD40-repeat36, have been identified for POAG. Among them, optineurin is a gene that links particularly to normal pressure glaucoma, a subtype of POAG that accounts for approximately 30% of the POAG cases [6]. In this condition, the glaucomatous damage occurs with the intraocular pressures within the normal limits, although the progression of the damage is believed to be still IOP dependent [6]. Optineurin mutations were noted to vary with ethnic background [7]. Mutations including Glu^50^→Lys (E50K), Met^98^→Lys (M98K), and Arg^545^→Gln (R545Q) in optineurin have been reported. The E50K mutation, found in Caucasian and Hispanic populations [7], seems to be associated with a more progressive and severe disease [8].

The human optineurin gene encodes a protein that contains multiple coiled-coil domains, at least one leucine zipper (amino acids 143–164) and a C-terminal zinc finger [9]. The optineurin protein from different species has high amino acid homology and the amino acid residue 50 glutamic acid is conserved [10]. Optineurin is ubiquitously expressed in non-ocular tissues such as the heart and brain [9] and in ocular tissues including the retina, trabecular meshwork (TM), retinal pigment epithelium (RPE) [10], and non-pigmented ciliary epithelium [10], [11]. RGCs are immunolabeled with a high intensity [12].

Optineurin shares a 53% amino acid homology with NEMO (NF-κB essential modulator) and was identified as a NEMO-related protein [13]. Recently, optineurin has been shown to be a negative regulator of NF-κB [14]. Like NEMO, the C-terminal half of the optineurin sequence binds K-63 linked polyubiquitinated chains [15]–[17].

Optineurin has also been demonstrated to interact with transcription factor IIIA [18], Rab8 [12], [19]–[22], huntingtin [19], myosin VI [20], [21], metabotropic glutamate receptor [23], and TANK-binding kinase 1 [24]. In optineurin-overexpressing human RPE and TM cells, granular structures or dots referred to as foci that formed near the nucleus are noted to partially colocalize with transferrin receptor (TfR) [25]. Rab8, huntingtin, myosin VI, and TfR are all players in membrane trafficking [26]–[28], and optineurin has also been implicated in protein trafficking [17], [20], [22].

Protein trafficking by the endocytic pathway is an essential cellular mechanism critical for many functions including cell signaling, nutrient acquisition and maintenance of the plasma membrane [29]. The uptake route is composed of organelles, known as endosomes that communicate in a unidirectional manner from early to late endosomes to lysosomes. The best characterized endocytic pathway is clathrin-dependent endocytosis that mediates the constitutive uptake of ligands such as transferrin [30].

Previously, studies using small interfering RNA (siRNA) have suggested that optineurin is involved in the exocytosis of vesicular-stomatitis-virus G protein [20]. Here we undertook an investigation to determine in RPE cells and a model of RGC [31], RGC5 cell line [32], the effects of optineurin overexpression and downregulation as well as E50K mutation on the internalization of transferrin (Tf), which has been extensively used as a marker for receptor-mediated endocytosis via clathrin-coated pits [33]. While RGC is a cell type of direct relevance to glaucoma, RPE cells are not currently implicated in any forms of glaucoma. They were chosen for the study largely based on the finding that optineurin is prominently expressed in the RPE tissue in mouse and monkey eyes [10], [34]. In both RPE and RGC5 cells, the possible association between optineurin and markers of vesicular trafficking compartments was assessed. In addition, biochemical interactions between optineurin and various small GTPase Rab proteins, active protagonists in the transport of macromolecules along the endocytic pathways, and TfR, were determined by co-immunoprecipitation (IP).

## Results

### Reduction of Tf uptake by expression of wild type optineurin-green fluorescence protein (OPTN_WT_-GFP)

**Figure 1:**
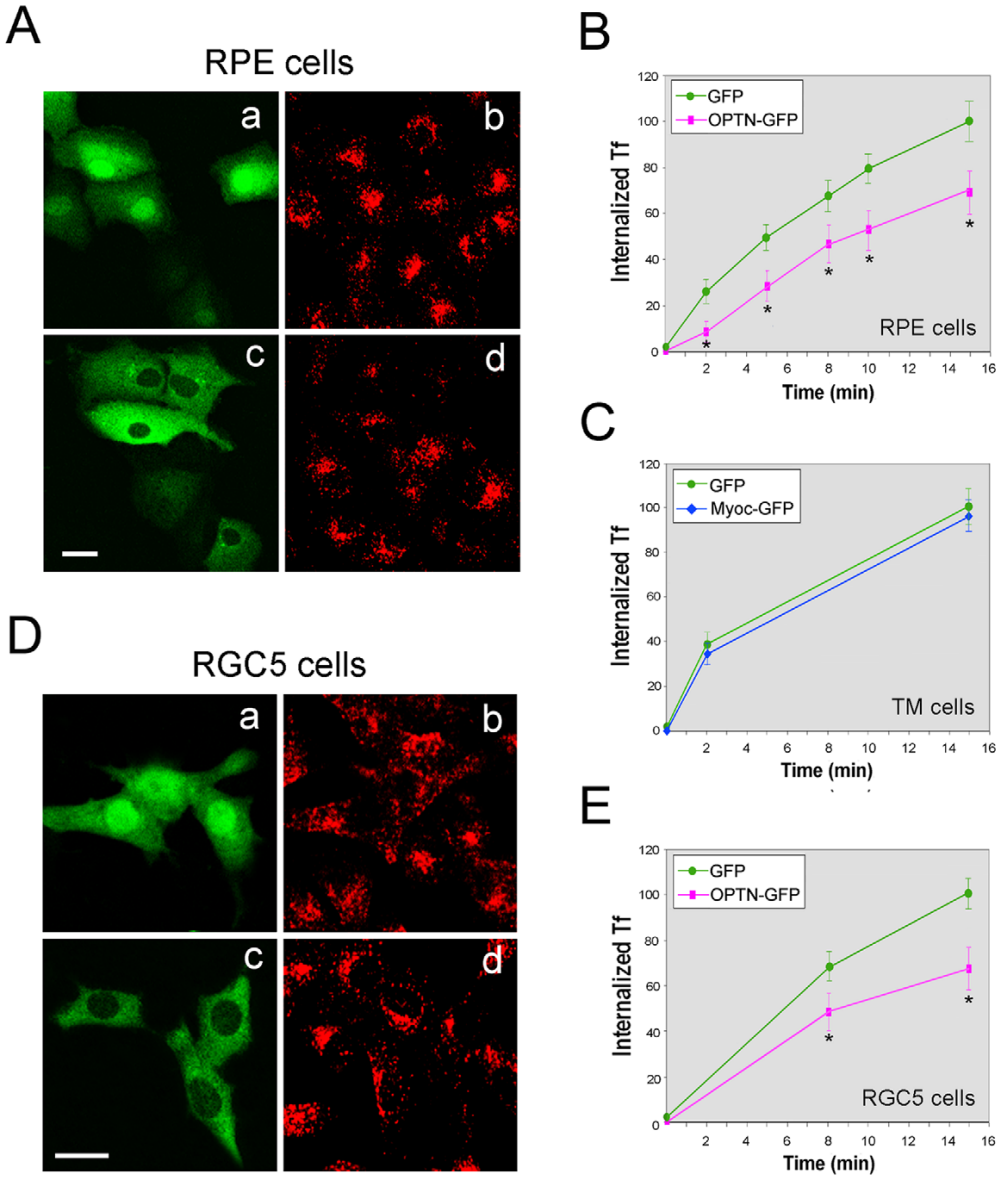
Overexpression of optineurin-GFP (OPTN_WT_-GFP) but not myocilin-GFP (MYOC-GFP) impedes transferrin (Tf) uptake. (**A**) Fluorescent images of GFP (Aa and Ab)- and OPTN_WT_-GFP (Ac and Ad)-expressing RPE cells after incubation with Texas red-Tf (TR-Tf) for 15 min. The cells were fixed and the fluorescent images were captured under a Leica confocal microscope. Transfected cells (green) are seen in Aa and Ac and the internalized TR-Tf (red) is seen in Ab and Ad. Scale bar, 20 µm. (**B**) Quantification of TR-Tf uptake in GFP (green filled circles)-, and OPTN_WT_-GFP (pink squares)-expressing RPE cells. Fluorescence intensity of TR-Tf in the cells was measured at 0-, 2-, 5-, 8-, 10-, and 15-min time points. Images in seven randomly selected 20× fields, each containing at least 8 transfected cells, were analyzed. TR-Tf internalization for samples at each time point is expressed as percentage relative to that of the 15-min uptake in GFP-expressing mock control cells. Data presented are mean ± standard deviation (SD) from a representative of three independent experiments. *, P<0.0001 (n = 8) compared to GFP controls. (**C**) Quantification of TR-Tf uptake in GFP (green filled circles)-, or MYOC-GFP (blue diamonds)-expressing TM cells. (**D**) Fluorescent images of GFP (Da and Db)- and OPTN_WT_-GFP (Dc and Dd)-expressing RGC5 cells (green) after incubation with TR-Tf (red, Db and Dd) for 15 min. Scale bar, 20 µm. (**E**) Quantification of TR-Tf uptake in GFP (green filled circles)-, and OPTN_WT_-GFP (pink squares)-expressing RGC5 cells. Fluorescence intensity of TR-Tf in the cells was measured at the 0-, 8-, and 15-min time points. The uptake is expressed as percentage (mean ± SD) relative to that of the 15-min uptake in GFP-expressing control cells. Experiments were repeated at least three times with similar results. *, P<0.001 compared to GFP controls.

We previously reported that the optineurin foci formed upon forced expression of OPTN_WT_-GFP in the perinuclear region, colocalizing with a population of TfR, a marker for endosomal vesicles [25]. To determine whether the overexpression affects Tf uptake, human RPE cells transfected with pOPTN_WT_-GFP or pEGFP-N1 (a plasmid vector used as a mock control here; genes can be cloned into this vector to be expressed as fusions to the N-terminus of GFP, for example, optineurin-GFP) were incubated with Texas red-conjugated Tf (TR-Tf) at 37°C. After 15 min, intense accumulation of red-fluorescent TR-Tf in perinuclear areas were seen in nontransfected (non-green) and GFP mock-transfected green cells (Fig. 1Aa and Ab). By comparison, the Tf accumulation in OPTN_WT_-GFP-expressing cells (Fig. 1Ac and Ad) was much reduced.

The Tf uptake in RPE cells was quantified. Six time points (0, 2, 5, 8, 10 and 15 min; Fig. 1B) were examined. The Tf uptake in pEGFP-N1-transfected cells was similar to that in nontransfected cells during the entire time course, indicating that GFP mock transfection did not affect the Tf internalization kinetics. On the other hand, the Tf uptake was significantly decreased (P<0.0001) in pOPTN_WT_-GFP-transfected cells. The reduction of Tf uptake was observed from the initial 2- and 5-min time points (Fig. 1B). For comparison purposes, RPE and TM cells were transfected to overexpress wild type myocilin-GFP [35] and its influence on Tf uptake was investigated. Unlike optineurin, myocilin did not exhibit any effect on the kinetics of Tf internalization (Fig. 1C, data shown were from TM cells). The inhibition of Tf uptake upon transfection with pOPTN_WT_-GFP in RPE cells was likewise observed in RGC5 cells (Figs. 1D and 1E).

### Lack of association between optineurin foci and endocytic markers on the Tf uptake route

**Figure 2:**
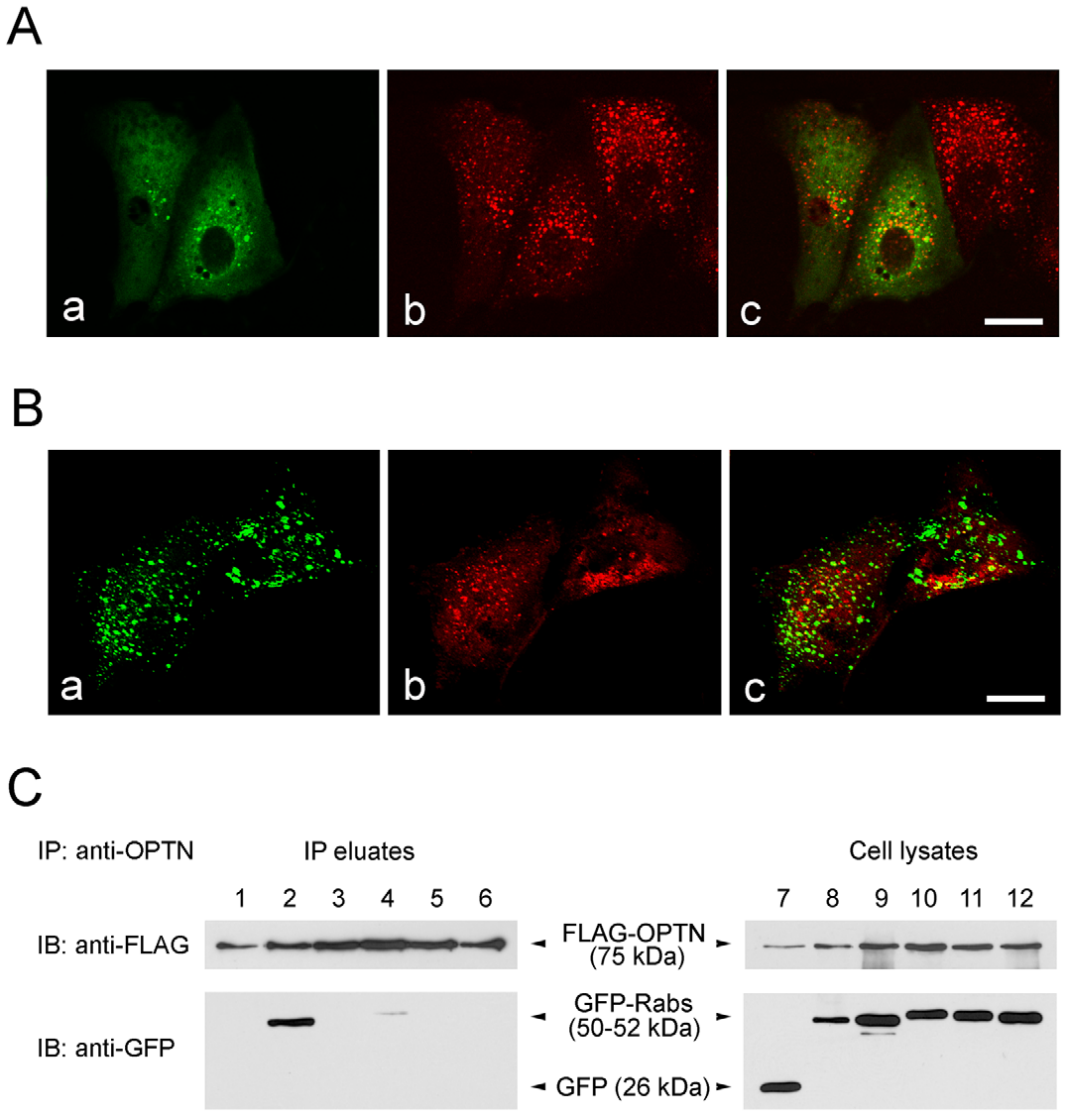
Lack of interactions between wild type optineurin (OPTN_WT_) and endocytic markers on the Tf uptake route. (**A**) RPE cells transfected to express OPTN_WT_-GFP (Aa, green) were immunostained with anti-EEA1 (Ab, red). (**B**) RPE cells co-transfected with pTarget-FLAG-OPTN_WT_ and pGFP-Rab5_WT_ (Ba, green) for 16 h were immunostained with anti-FLAG antibody (Bb, red). Merged images are presented in Ac and Bc. Optineurin foci largely did not colocalize with EEA1 (**A**) or Rab5 (**B**). Scale bar, 20 µm. (**C**) Co-immunoprecipitation (IP) of optineurin with Rab proteins. RPE cells expressing FLAG-OPTN_WT_ along with GFP (lanes 1 and 7), GFP-Rab8 (lanes 2 and 8), GFP-Rab4 (lanes 3 and 9), GFP-Rab5 (lanes 4 and 10), GFP-Rab11 (lanes 5 and 11), or GFP-Rab7 (lanes 6 and 12) were immunoprecipitated with anti-optineurin. The resulting IP eluates (lanes 1–6) were immunoblotted (IB). The 75-kDa FLAG-OPTN and 50–52-kDa GFP-Rabs were detected by anti-FLAG and anti-GFP antibodies, respectively. Thirty micrograms of cell lysates (lanes 7–12) were analyzed with the same antibodies to evaluate the expression level of fusion proteins.

The TfR-Tf complex is internalized through clathrin-coated pits, transported to peripheral early/sorting endosomes and then recycled back to cell surface either directly or via the pericentriolar recycling endosomes. RPE cells were transfected with pOPTN_WT_-GFP and immunostained for endosomal marker protein (Fig. 2A) or co-transfected with pTarget-FLAG-OPTN_WT_ and pGFP-Rabs to co-express endocytic markers (Fig. 2B). The molecules investigated included EEA1 (early endosome antigen-1) (Fig. 2A), an early endosome marker, Rab5 (Fig. 2B), a protein that controls the initial transport of Tf to early endosomes, Rab4, a marker found in both early and recycling endosomes, and Rab7, a marker for late endosomes. Results showed that there was no major colocalization with any of these marker proteins (Figs. 2A and B, Rab4 and Rab7 data not shown).

Co-IP was subsequently performed. RPE cells co-transfected with pTarget-FLAG-OPTN and pEGFP-N1, pGFP-Rab8, pGFP-Rab4, pGFP-Rab5, pGFP-Rab11 or pGFP-Rab7 were lysed and immunoprecipitated with anti-optineurin. Plasmids pEGFP-N1 and pGFP-Rab8 were included as a negative and a positive control, respectively. The resulting precipitates were subjected to immunoblotting. An anti-FLAG antibody detected the 75-kDa FLAG-optineurin band in all IP products (Fig. 2C, lanes 1–6), verifying that IP was properly performed. When anti-GFP antibody was used, only GFP-Rab8 band was obtained (Fig. 2C, lane 2), indicating that optineurin co-precipitated with GFP-Rab8 but not with any other Rabs. It was not due to poor expression of GFP-Rabs, because anti-GFP detected a fairly high amount of GFP or GFP-Rab proteins in total cell lysates (Fig. 2C, lanes 7–12). This suggested that optineurin had minimal association with proteins such as Rab5 that have active roles in Tf endocytosis.

### Rescue of Tf uptake by co-transfection with TfR but not with myosin VI and Rab8 constructs

**Figure 3:**
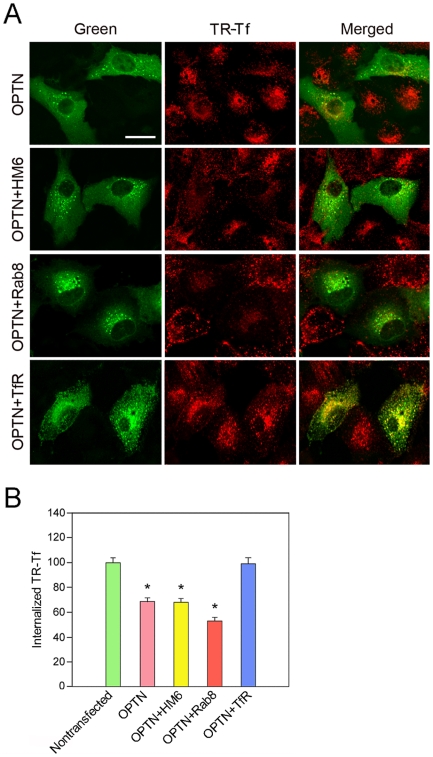
Rescue of Tf uptake by co-transfection with TfR but not with myosin VI and Rab8 constructs in RPE cells. (**A**) Fluorescent images of RPE cells. The cells transfected with pOPTN_WT_-GFP (OPTN) alone, or co-transfected with pOPTN_WT_-GFP and pMyoVI-EGFP (OPTN + HM6), pRab8_Q67L_-EGFP (OPTN + Rab8), or pTfR-EGFP (OPTN + TfR) were incubated with Texas Red-Tf (TR-Tf) for 15 min. The single or co-transfected cells are in green and the internalized TR-Tf is seen in red. The yellow staining in perinuclear regions (bottom panel, merged) indicated interaction and colocalization between the expressed TfR-GFP and the internalized TR-Tf. Scale bar, 20 µm. (**B**) Quantification of TR-Tf uptake in nontransfected and single- or co-transfected RPE cells. Note that co-transfection with myosin VI and Rab8 constructs had little effect, while co-expression of TfR restored the Tf uptake to the nontransfected control level in OPTN_WT_-GFP-expressing cells. Data are expressed as mean ± SD. Experiments are repeated at least three times, yielding similar results. *, P<0.001 compared to nontransfected cells.

To determine whether the impairment of Tf uptake could be rescued by optineurin binding proteins myosin VI, Rab8, or TfR, RPE (Fig. 3) and RGC5 (data not shown) cells were co-transfected with pOPTN_WT_-GFP along with pMyoVI-EGFP, pRab8_Q67L_-EGFP, or pTfR-EGFP [36] for 16 h. They were then incubated with TR-Tf and the Tf uptake was quantified. It was found that co-transfection with myosin VI and Rab8 constructs (Fig. 3A) had little effect, while co-expression of TfR expression plasmid (Fig. 3A) restored the Tf uptake to the nontransfected control level in OPTN_WT_-GFP-expressing cells (Fig. 3B). The yellow staining in perinuclear regions (Fig. 3A, bottom panel, merged) indicated interaction and colocalization between the expressed TfR-GFP and the internalized TR-Tf.

### Total TfR level was not, but TfR distribution was altered by OPTN_WT_-GFP expression

**Figure 4:**
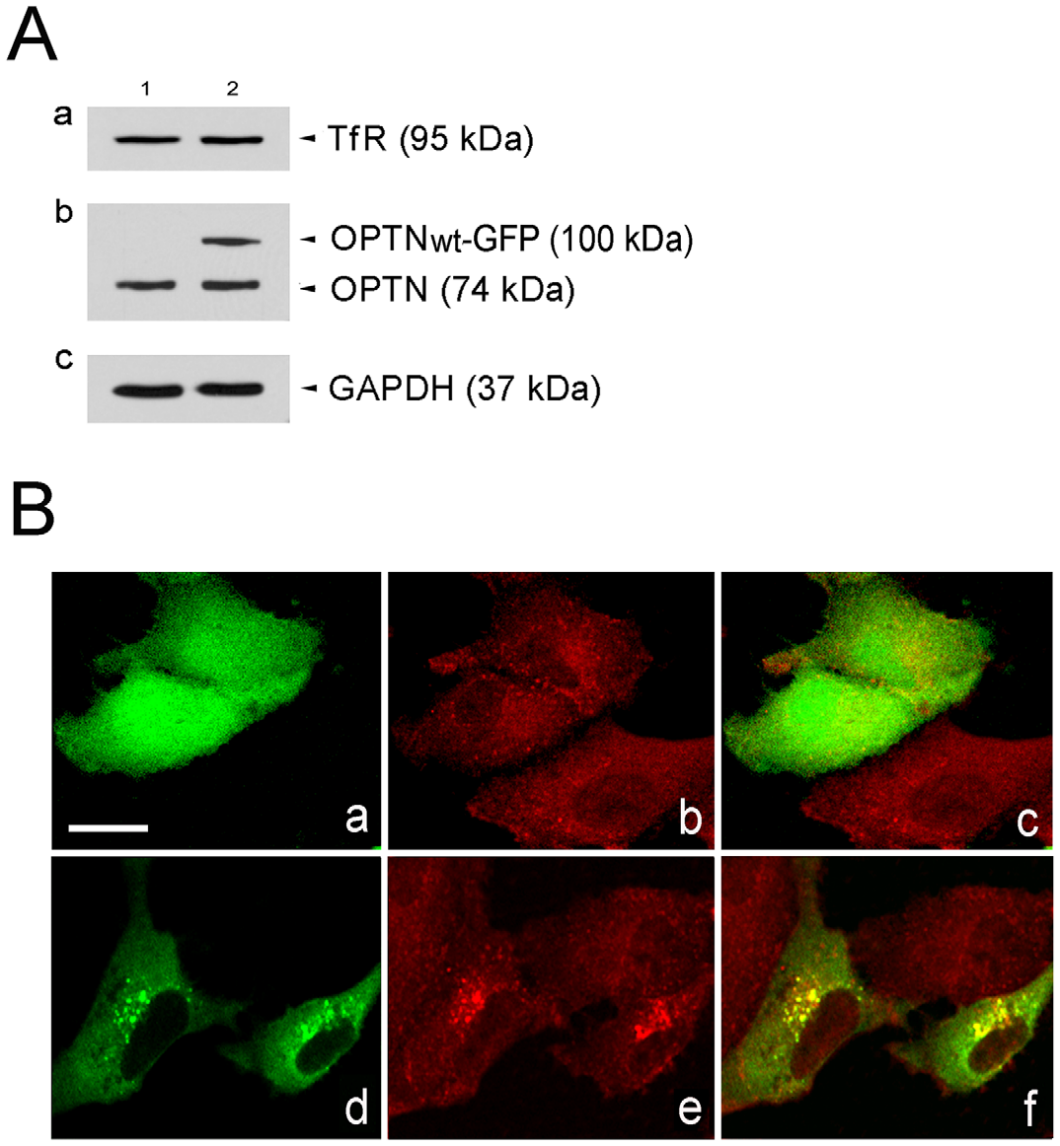
Effects of optineurin overexpression on the total level and distribution of the endogenous TfR in RPE cells. (**A** )Lysates (50 µg total protein) from cells transfected with pEGFP-N1 (lane 1) or pOPTN_WT_-GFP (lane 2) were immunoblotted with anti-TfR (Aa), anti-optineurin (OPTN) (Ab), and anti-GAPDH (Ac) antibodies. The expression level of 95-kDa TfR was not reduced by OPTN_WT_-GFP overexpression. Anti-OPTN detected the 74-kDa endogenous OPTN band in both lanes and a 100-kDa OPTN_WT_-GFP band in lane 2. Anti-GAPDH that detected a 37-kDa band, was used to control protein loading. (**B**) Distribution of TfR in GFP- and OPTN_WT_-GFP-expressing cells. RPE cells transfected with pEGFP-N1 (Ba-Bc, green) and pOPTN_WT_-GFP (Bd-Bf, green) were immunostained with anti-TfR (Bb, Bc, Be, Bf, red). Merged images are shown in Bc and Bf. Colocalization between OPTN_WT_-GFP and TfR is seen in yellow (Bf).

The TfR protein level in pEGFP-N1 (mock control)- or pOPTN_WT_-GFP-transfected RPE cells was examined by Western blotting. The glyceraldehyde 3-phosphate dehydrogenase (GAPDH) level was used as a protein loading control. In addition, the level of the endogenous optineurin and the OPTN_WT_-GFP fusion protein was also determined to confirm that the cells were indeed transfected. Results in Fig. 4A showed that the TfR level in cells expressing OPTN_WT_-GFP was similar to that of the mock control. The reduction of Tf uptake in pOPTN_WT_-GFP-transfected cells was thus unrelated to the expression level of total TfR.

In GFP-expressing as well as nontransfected cells, punctate TfR staining was seen distributing largely diffusely in the cytoplasm (Fig. 4Bb). After optineurin transfection, TfR was observed to become more concentrated in the perinuclear region, colocalizing at least partially with the optineurin foci (Fig. 4Be and f). It appeared that TfR was recruited and sequestered in the perinuclear area by the optineurin foci.

### Tf uptake, total TfR level and TfR distribution in cells expressing OPTN_E50K_-GFP

**Figure 5:**
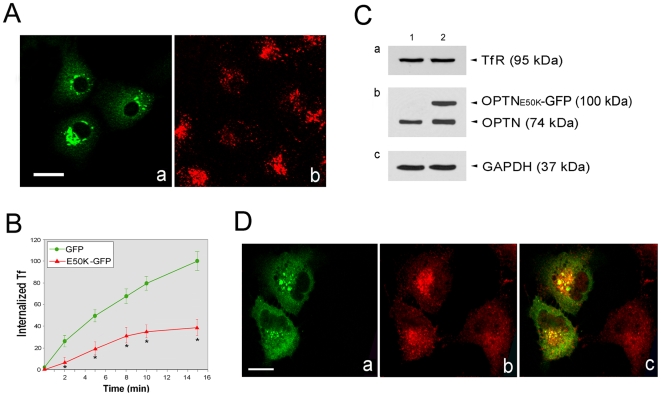
Effects of OPTN_E50K_-GFP expression on Tf uptake, total TfR level, and TfR distribution in RPE cells. (**A**) Fluorescent images of RPE cells transfected with pOPTN_E50K_-GFP for 16 h and incubated with TR-Tf for 15 min. The transfected cells are in green (Aa) and the internalized TR-Tf is seen in red (Ab). Scale bar, 20 µm. (**B**) Quantification of TR-Tf uptake in GFP (green filled circles)- and OPTN_E50K_-GFP (red triangles)-expressing cells. Fluorescence intensity of TR-Tf in the cells was measured at various time points. *, P<0.0001 compared to GFP controls. (**C**) Lysates (50 µg total protein) from RPE cells transfected with pEGFP-N1 (lane 1) or pOPTN_E50K_-GFP (lane 2) were immunoblotted with anti-TfR (Ca), anti-optineurin (OPTN) (Cb), and anti-GAPDH (Cc) antibodies. The expression level of 95-kDa TfR was similar in lanes 1 and 2. A 74-kDa endogenous optineurin band and a 100-kDa OPTN_E50K_-GFP band were detected. The GAPDH level was used to control protein loading. (**D**) RPE cells transfected with pOPTN_E50K_-GFP (Da, green) were immunostained with anti-TfR (Db, red). Merged image (Dc) shows colocalization between OPTN_E50K_-GFP and TfR in yellow.

We have shown that overexpression of a disease-causing mutant optineurin, OPTN_E50K_, induced foci formation more prominently than that seen in the wild type [25]. Here, experiments were carried out to evaluate whether E50K expression affects the Tf uptake. As seen in Fig. 5A, the TR-Tf accumulation within 15 min in OPTN_E50K_-GFP-expressing cells was much reduced compared to that in the neighboring nontransfected and GFP control (data not shown) cells. Quantitative analyses (Fig. 5B) showed that the TR-Tf uptake was significantly (P<0.0001) reduced and the reduction was more pronounced in cells expressing OPTN_E50K_-GFP (approximately 70–80% reduction) than OPTN_WT_-GFP (approximately 30–40% reduction, Fig. 1B).

Forced expression of OPTN_E50K_-GFP, similar to that seen with the wild type (Figs. 4A and 4B), did not alter the total level of TfR (Fig. 5C), but changed the TfR localization to a more perinuclear pattern (Fig. 5D). An enhanced colocalization between the OPTN_E50K_ foci and TfR was observed (Fig. 5D).

### Tf uptake, total TfR level and TfR distribution in cells expressing OPTN_L157A_-GFP

**Figure 6:**
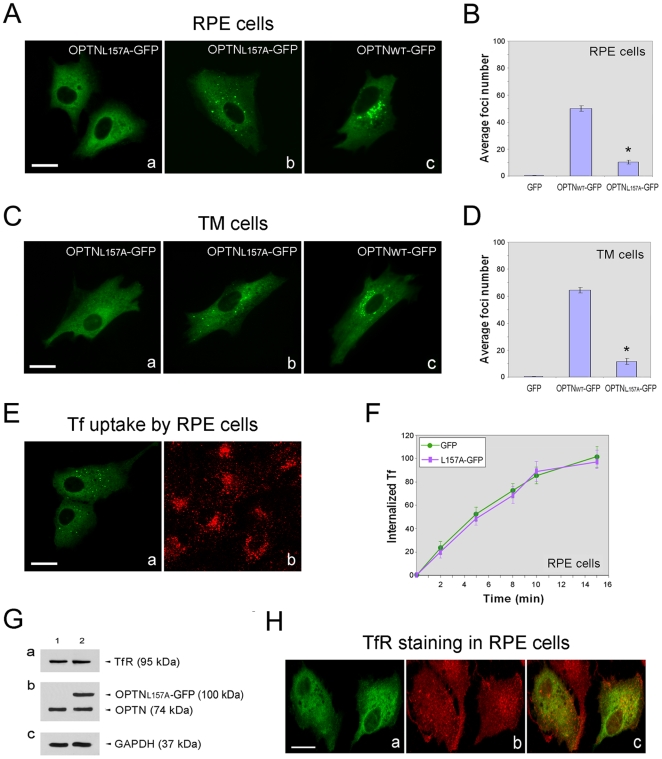
Cellular localization of OPTN_L157A_-GFP and effects of OPTN_L157A_-GFP expression on Tf uptake, total TfR level, and TfR distribution. (**A**) RPE cells were transfected with pEGFP-N1 (GFP mock control, not shown), pOPTN_L157A_-GFP (a and b) or pOPTN_WT_-GFP (c) for 16 h. Representative confocal images shown in Aa and Ab depict two major patterns of OPTN_L157A_ distribution. Scale bar, 20 µm. (**B**) The number of foci in at least 60 RPE transfectants was counted and the average number of foci per transfectant is shown. Data (mean ± SD) presented are representative from three independent experiments. *, P<0.0001 compared to OPTN_WT_-GFP-expressing cells. (**C**) Human TM cells were transfected pEGFP-N1 (mock control, not shown), pOPTN_L157A_-GFP (a and b) or pOPTN_WT_-GFP (c). Two patterns of OPTN_L157A_ distribution are seen in a and b. Scale bar, 20 µm. (**D**) The number of foci in TM transfectants was counted. The average number of foci per transfectant (mean ± SD) is presented. *, P<0.0001 compared to OPTN_WT_-GFP-expressing cells. (**E**) Fluorescent images of OPTN_L157A_-GFP-expressing RPE cells after internalization of TR-Tf for 15 min. The transfected cells are in green (Ea) and the internalized TR-Tf is shown in red (Eb). Scale bar, 20 µm. (**F**) Quantification of TR-Tf uptake in OPTN_L157A_-transfected RPE cells. Fluorescence intensity of TR-Tf, determined as in Fig. 1B, is expressed as percentage relative to that of the 15-min uptake in GFP-expressing controls. Data (mean ± SD) presented are representative of three independent experiments. (**G**) Lysates (50 µg total protein) from RPE cells transfected with pEGFP-N1 (lane 1) or pOPTN_L157A_-GFP (lane 2) were immunoblotted with anti-TfR (Ga), anti-optineurin (OPTN) (Gb), and anti-GAPDH (Gc, used to control protein loading) antibodies. Total level of the 95-kDa TfR was similar in lanes 1 and 2. A 74-kDa endogenous optineurin band and a 100-kDa OPTN_L157A_-GFP band were detected. (**H**) RPE cells transfected with pOPTN_L157A_-GFP (Ha, green) were immunostained with anti-TfR (Hb, red). The diffuse distribution of OPTN_L157A_-GFP overlapped with that of TfR in yellow in the merged image (Hc).

Leucine zipper motif is known to be sites for protein-protein interactions. Optineurin protein contains one leucine zipper domain (amino acid residues 143–164) at the N-terminus and Rab8 has been suggested to interact with optineurin through the N-terminal region (amino acid residues 141–209) that includes the leucine zipper domain [12], [19]–[22], [37]. To obliterate this domain, we introduced a point mutation by in vitro mutagenesis to substitute Leu^157^ to Ala (L157A) in the optineurin sequence. OPTN_L157A_ construct in pEGFP vector (pOPTN_L157A_-GFP) was made. When transfected, two patterns of OPTN_L157A_-GFP distribution were noted in human RPE (Fig. 6A), TM (Fig. 6C), and RGC5 (data not shown) cells. In about 60–70% of transfected cells, foci formation was hardly seen and the OPTN_L157A_-GFP fusion protein distributed diffusely throughout the cytoplasm (Fig. 6Aa and 6Ca). Foci were observed in the remaining cells but they were not concentrated in the perinuclear area (Fig. 6Ab and 6Cb). Compared to those seen in wild type optineurin-expressing cells, the OPTN_L157A_ foci appeared to be fewer in number, smaller in size, and more dispersed into the cytoplasm. Quantitative counting confirmed the microscopic observations that cells transfected with pOPTN_L157A_-GFP had on average a lower number of foci compared with those transfected with pOPTN_WT_-GFP (10.3±11 versus 50.3 ±1,9 in RPE cells, P<0.0001, Fig. 6B; 11.5±2.3 versus 64.6±2.1 in TM cells, P<0.0001, Fig. 6D).

OPTN_L157A_-GFP-Expressing RPE cells were incubated with TR-Tf to assess the Tf uptake. As shown in Fig. 6E, the fluorescence intensity of TR-Tf after a 15-min uptake was similar in cells expressing OPTN_L157A_-GFP and nontransfected cells. Quantitative analyses verified that the Tf uptake was unaltered by the L157A mutation compared to GFP controls (Fig. 6F). Furthermore, overexpression of OPTN_L157A_-GFP did not vary the total level (Fig. 6G), nor the largely diffuse cytoplasmic distribution pattern (Fig. 6H) of TfR.

### Self binding of optineurin mutants and interactions with Rab8 and TfR

**Figure 7:**
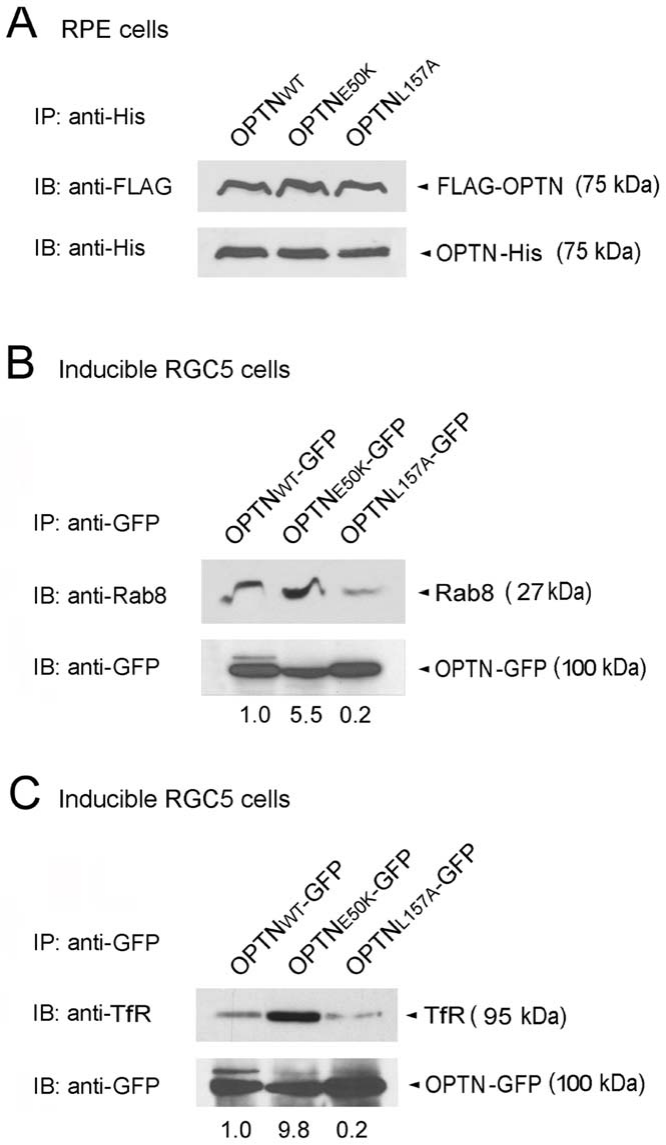
Self binding of optineurin mutants and their interactions with Rab8 and TfR. (**A**) Self binding of wild type optineurin and mutants. RPE cells were co-transfected with pOPTN_WT_-His + pTarget-FLAG-OPTN_WT_, pOPTN_E50K_-His + pTarget-FLAG-OPTN_E50K_, or pOPTN_L157A_-His + pTarget-FLAG-OPTN_L157A_ for 16 h. Cell lysates were immunoprecipitated (IP) with polyclonal anti-His. The IP eluates were immunoblotted (IB) with monoclonal anti-FLAG or monoclonal anti-His. The anticipated molecular weight of the proteins detected by both antibodies was 75 kDa. No protein band was detected when rabbit IgG was used for immunoprecipitation (data not shown). (**B**) Binding of wild type optineurin and mutants with endogenous Rab8. Inducible RGC5 cells were treated with doxycycline for 16 h to express OPTN_WT_-GFP, OPTN_E50K_-GFP and OPTN_L157A_-GFP. Cell lysates were immunoprecipitated (IP) with polyclonal anti-GFP. The IP eluates were immunoblotted (IB) with monoclonal anti-Rab8 or polyclonal anti-GFP. The intensity ratio between the detected 27-kDa-Rab8 band and the 100 kDa-OPTN-GFP band relative to that of the wild type optineurin is presented. No protein band was observed in lysate samples obtained from non-induced cells (data not shown). (**C**) Binding of wild type optineurin and mutants with endogenous TfR. Inducible stable cell lines were induced by doxycycline to express OPTN_WT_-GFP, OPTN_E50K_-GFP or OPTN_L157A_-GFP. Cell lysates were immunoprecipitated (IP) with a polyclonal anti-GFP antibody. The IP eluates were immunoblotted (IB) with a monoclonal anti-TfR antibody or anti-GFP. The intensity ratio between the 95-kDa TfR band and the 100 kDa-OPTN-GFP band relative to that of the wild type optineurin is presented. No protein band was observed in lysate samples obtained from non-induced cells (data not shown).

Our laboratory has previously reported that wild type optineurin interacts with itself to form homo-hexamer as well as with its binding partners to form supermolecular complexes [36]. The foci formation is presumably related to these complex formations. To determine whether a point mutation of optineurin at amino acid residue 50 from Glu to Lys or residue 157 from Leu to Ala would change its ability to self interact, RPE cells were co-transfected with pOPTN_E50K_-His + pTarget-FLAG-OPTN_E50K_ and pOPTN_L157A_-His + pTarget-FLAG-OPTN_L157A_ for 16 h. Cells were also co-transfected with pOPTN_WT_-His + pTarget-FLAG-OPTN_WT_ as a positive control. The lysates were immunoprecipitated with a polyclonal anti-His antibody and the IP eluates were immunoblotted with a monoclonal anti-FLAG antibody (Fig. 7A, top panel). The anticipated molecular weight of the proteins detected by anti-FLAG antibody was 75 kDa. The membrane was also immunoblotted with anti-His monoclonal antibody to verify that IP was properly performed (Fig. 7A, lower panel). The results revealed that both E50K and L157A mutants were still capable of binding to themselves and the binding was comparable to that of the wild type optineurin. Native blue gel electrophoresis performed further detected formation of the 420 kDa homo-hexamer by the wild type and the two optineurin mutants (data not shown).

Rab8 has been suggested to bind to optineurin through the leucine zipper-containing N-terminal region [12], [19]–[22], [37]. To determine whether E50K or L157A mutation affects the Rab8 binding, tetracycline-regulated (Tet-on) inducible stable RGC5 cell lines [36] established in our laboratory were used. These cells, treated with doxycycline for 16 h to induce the expression of E50K-, L157A-, as well as wild type optineurin-GFP [36], offered an advantage that the expression of fusion protein was essentially uniform in culture plates. The level of induced gene expression could also be specified (for example, 10–15 fold increase relative to the endogenous level, as in our experiments) from selected clones. By contrast, cell transfection, depending on transfection efficiency, generally yielded only a population of transfected cells, complicating quantitative assessments. The level of transgene expression was also difficult to regulate.

Co-IP was performed using anti-GFP antibody and the precipitated proteins were probed with anti-Rab8 to detect the pull down of the endogenous Rab8 (Fig. 7B, upper panel). Anti-GFP probing was also used to verify the IP procedure and to determine the expression level of GFP fusion proteins (Fig. 7B, lower panel). Results obtained disclosed that the 27-kDa Rab8 was present in all three pull downs. The level of Rab8 immunoprecipitated by anti-GFP from the lysate of OPTN_E50K_-GFP-expressing cells was approximately 5 fold higher than that from OPTN_WT_-GFP-expressing cells, indicating a stronger Rab8 binding by the E50K mutant. The Rab8 binding however was reduced by approximately 5 fold by the L157A mutation. These data suggested that the leucine zipper domain in optineurin is at least partially required for Rab8 binding.

The inducible RGC5 cell lines were similarly used to provide quantitative assessment of the TfR binding capability of wild type and mutant optineurin. Cell lysates were immunoprecipitated with polyclonal anti-GFP, and the IP eluates were immunoblotted with mouse anti-TfR to detect the endogenous TfR. Again, the E50K mutant bound approximately 10-fold higher, while the L157A one bound roughly 5-fold lower, amounts of endogenous TfR than the wild type optineurin (Fig. 7C, top panel). The same membrane was also immunoblotted with anti-GFP (Fig. 7C, lower panel).

### Level of TfR on the cell surface

**Figure 8:**
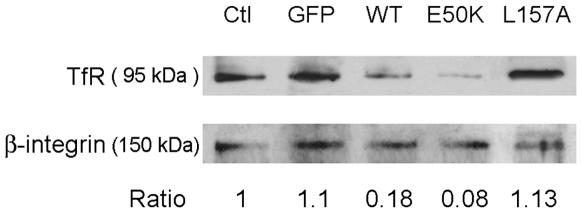
Overexpression of wild type and E50K optineurin, but not L157A mutant, alters the surface TfR level in RPE cells. Cells were transfected to express GFP, as well as wild type (WT)-, E50K-, and L157A-optineurin-GFP. Surface TfR was detected by Western blotting with anti-TfR after surface protein biotinylation and isolation of biotinylated proteins. The level of surface TfR was normalized to that of a well known cell surface protein, β1-integrin. The ratios of TfR to β1-integrin are presented. Ctl, nontransfected control RPE cells.

To provide further evidence that the sequestration of TfR by the optineurin foci may be an important factor in blocking of the Tf uptake, the level of surface TfR was determined. The surface proteins in transfected RPE cells were labeled with non-permeable sulfo-NHS-SS-biotin reagent as described previously [38]. Results (Fig. 8) showed that relative to a well-known surface protein, β1-integrin, the surface TfR level in OPTN_WT_-GFP and OPTN_E50K_-GFP overexpressing cells was markedly decreased than that in GFP controls. Transfection with pOPTN_L157A_-GFP, on the other hand, did not lower the TfR level on the cell surface.

### Optineurin knock down by siRNA compromises the Tf uptake

**Figure 9:**
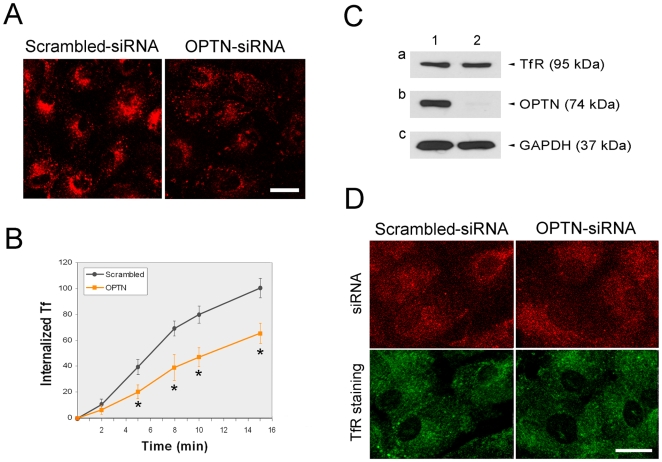
Knockdown of the endogenous optineurin inhibits Tf uptake but has little effect on the TfR level or distribution in RPE cells. (**A**) Fluorescence images of RPE cells transfected for 48 h with 50 nM generic scrambled control or optineurin (OPTN) siRNA after a 15-min uptake of TR-Tf (in red). Scale bar, 20 µm. (**B**) Quantification of Tf uptake in scrambled (dark gray filled circles)- and OPTN siRNA-(orange squares) transfected cells. Fluorescence intensity of 7 randomly selected 20× fields at 0-, 2-, 5-, 8-, 10-, and 15-min time points was measured and divided by the number of cells. The internalized TR-Tf at each time point was expressed as percentage relative to that of the 15-min uptake in scrambled siRNA-transfected cells. Data (mean ± SD) presented are representative of three independent experiments. *, P<0.0001 compared to scrambled controls. (**C**) Expression level of endogenous TfR and optineurin in optineurin siRNA-treated cells. The cell lysates were immunoblotted with anti-TfR, anti-optineurin (OPTN), and anti-GAPDH. The TfR level was not altered while the optineurin knockdown (lane 2) was confirmed. Anti-GAPDH antibody was used to assess protein loading. (**D**) RPE cells were transfected with scrambled or OPTN siRNA along with Cy3-labeled control siRNA. Cy3 (red) fluorescence was seen in nearly all cells (top panel), indicating that the siRNA transfection efficiency was >95%. These cells were immunostained with anti-TfR (green, bottom panel) for TfR distribution. No alteration was observed. Scale bar, 20 µm.

Scrambled (mock control) or optineurin siRNA was transfected into RPE cells for 2 days. These cells were then incubated with TR-Tf. Fluorescent microscopy showed that the amount of Tf internalized after 15 min in optineurin-depleted cells was considerably lower than that in mock controls (Fig. 9A). The fluorescence intensity, measured at 5 different time points (2, 5, 8, 10 and 15 min), was significantly (P<0.045 for the 2-min time point, P<0.0001 for others) reduced in cells transfected with optineurin siRNA (Fig. 9B). The TR-Tf intensity in optineurin siRNA-treated cells was generally about 50–65% of that in scrambled controls. The expression level of optineurin was, as anticipated, downregulated in optineurin siRNA-transfected cells compared to controls (Fig. 9C), while the total TfR expression level was unchanged (Fig. 9C). Intracellular distribution of TfR was also similar in both scrambled and optineurin siRNA-treated cells (Fig. 9D).

## Discussion

Optineurin is a gene linked to glaucoma. The present study demonstrates that overexpression of wild type optineurin results in an impairment of the Tf uptake in both RPE and RGC5 cells (Fig. 1). Such a phenotype, interestingly, was not observed when the cells were transfected to overexpress myocilin, product of another glaucoma gene (Fig. 1C). These new findings support our previous conclusions [32], [35] that the two glaucoma genes have different functions and may contribute to the development of glaucoma via distinct mechanisms.

The uptake defect manifested by optineurin overexpression can be rectified by co-expression of TfR, but not by myosin VI or Rab8 (Fig. 3), all of which are optineurin-interacting proteins [17], [19], [25], [36], [37]. The new data suggest that TfR, but not myosin VI or Rab8, may be the key factor involved in the optineurin inhibitory phenotype. The total level of TfR is found maintained after optineurin transfection (Fig. 4A). However, TfR molecules that distribute diffusely in the cytoplasm are seen to become concentrated around the perinuclear region, colocalizing at least partially with the foci in transfected cells (Fig. 4B). More remarkably, the surface TfR level is substantially decreased (Fig. 8). It appears therefore that the TfR molecules, via interactions with optineurin, are recruited and sequestered by the foci formed near the perinuclear area (Fig. 7B). This arrest consequently leads to diminished availability of surface TfR and an impeded Tf uptake.

E50K is a mutation prevalent in patients with normal tension glaucoma [6], [8]. Our data, consistent with those published very recently [17], revealed that the E50K mutation, with an enhanced ability to interact with TfR (Fig. 7B), blocked the Tf uptake (Fig. 5A and 5B), modified the TfR distribution (Fig. 5D), and depleted the surface TfR (Fig. 8) even more dramatically than the wild type. This, along with the previous observations of a more prominent foci formation, more severe fragmentation of the Golgi complex [25], and a higher level of apoptosis [32] than the wild type, strongly indicates that E50K is a gain-of-function mutation. The PROSITE analysis [39] suggests that introduction of Glu^50^→Lys mutation results in no particular changes either in the structure or the conformation of optineurin (data not shown). Nevertheless, patients who have E50K mutation in clinical setting are reported to suffer glaucomatous defects more severe than those without this mutation [8].

L157A mutation has not been identified clinically in any patients to date and is more than likely not disease causing or related. Opposing to the E50K mutation, few foci were formed in cells expressing OPTN_L157A_-GFP (Fig. 6A–D) and the forced expression had no discernible effects on the Tf uptake (Fig. 6E and 6F). Perhaps due to the much decreased interaction with TfR (Fig. 7B), the TfR distribution (Fig. 6H) and the cell surface TfR level (Fig. 8) was unaffected by the L157A mutation. Taken all the data together, we conclude that the inhibition of Tf uptake triggered by overexpressed wild type and E50K optineurin is related to the interaction between optineurin and TfR, the recruitment and sequestration of TfR to the optineurin foci and the ensued attenuation of TfR molecules on the cell surface.

By computer analysis (http://2zip.molgen.mpg.de/), the change of Leu^157^ to Ala in the optineurin sequence may lead to obliteration of the leucine zipper (data not shown). The much reduced ability of L157A mutant to interact with Rab8 (Fig. 7B) and TfR (Fig. 7C) suggests that the optineurin association with Rab8 and TfR requires, at least in part, an intact leucine zipper motif. Since the ubiquitin binding domain (UBD) and the myosin VI and hungtintin interaction sites all reside in proximity to the C-terminus of the optineurin sequence [16], [17], neither the ubiquitination nor the aforementioned protein interactions should be impacted by the E50K or the L157A mutation. In support of this notion, both mutants, like the wild type optineurin, were found to be ubiquitinated (data not shown).

The foci formed in wild type and E50K optineurin-expressing cells are likely due to self binding of both the wild type and E50K molecules as well as their interactions with other proteins including Rab8 and TfR. Leucine zipper motif is known to be sites for protein-protein interactions. Since the L157A optineurin is still capable of interacting with itself (Fig. 7A), the lack of foci formation in L157A optineurin-GFP-expressing cells, in comparison to the wild type and the E50K counterparts, may be related to the perturbed binding of this mutant to Rab8 (Fig. 7B) and TfR (Fig. 7C) and perhaps other as-yet-unknown leucine zipper-binding protein(s) as well.

As stated above, our finding that E50K mutation results in a reduced uptake of Tf has been reported recently by Nagabhushana et al. [17]. Other consequences of E50K mutation [25,36, present study] such as the formation of foci (vesicular structures) and enhanced binding with Rab8 and TfR are also consistent with those descried previously [17]. The Tf uptake phenotype was not observed by Nagabhushana et al. [17] when the wild type optineurin was force expressed in HeLa cells, whereas it was seen in our hands using RPE and RGC5 cells. The disparity could be related to differences in cell types. Our study in addition provided quantitative data and biochemical evidence to substantiate the notion that the declined Tf uptake is related to the attenuated TfR level on the cell surface (Fig. 8).

Nagabhushana et al. in their report suggested that the UBD of optineurin is required for the formation of vesicular structures (foci), impairment of Tf uptake, and cell death [17]. Yet the current L157A findings indicate that the leucine zipper motif is also a requirement. As discussed above, the UBD located in the C-terminal region is intact in the L157A mutant, which was found ubiquitinated (data not shown). This mutant, similar to the ubiquitin-binding deficient but leucine zipper-intact D477N mutant [17], does not cause much foci formation (Fig. 6A–D) or induce apoptosis (data not shown) as was seen in the wild type and E50K optineurin cases. This argues for the conclusion that both the UBD at the C-terminus and the leucine zipper motif at the N-terminus are necessary requirements for the foci formation, Tf trafficking phenotype and apoptosis.

Using siRNA techniques, a previous study [10] showed that downregulation of optineurin reduced significantly the transport of vesicular-stomatitis-virus G protein to the cell surface [20] and altered the morphology of Golgi complex in normal rat kidney and HeLa cells. We have extended the investigation, demonstrating that knocking down optineurin compromised the Tf uptake. In addition, we showed that the optineurin depletion did not alter either the total TfR level or its distribution in cells.

As both the overexpression and the suppression of optineurin cause a reduction in the Tf internalization, a role of optineurin in this process is implicated. In the literature, there have been reports of various genes [40]–[45] displaying identical or similar phenotypes under both overexpression and depletion conditions. For example, inhibition of Tf internalization was seen to result from both overexpression and siRNA-mediated silencing of EHD2 (EH domain protein 2) [40]. Genes Rab22a [41], REP15 (Rab15 effector protein) [42], and Hrs (hepatocyte growth factor-regulated tyrosine kinase substrate) [43], [44] have also been noted to generate the same phenotype upon overexpression and knockdown: inhibited Tf recycling, inhibited TfR recycling, and impaired degradation of epidermal growth factor, respectively. In the budding yeast, overexpression and deletion of Bud2 both resulted in a significance increase in the rate of actomyosin ring contraction [45]. In this last example, Bud2 overexpression was thought to act as a dominant negative. In the others, it was speculated that overexpression may lead to formation of unbalanced protein complexes, trapping proteins associated with endocytosis steps and inhibiting in turn their normal functions. This appears to be the likely scenario involved in optineurin overexpression. Evidence is provided in the current study that the inhibition of Tf uptake by overexpressed optineurin is mediated largely through the foci formation and the augmented optineurin-TfR interaction. The precise mechanism involved in the depletion situation however still remains to be defined.

E50K produces a more prominent foci formation [17], [25], more severe fragmentation of the Golgi complex [25], and a higher level of apoptosis [32] than overexpression of the wild type optineurin. Representing a gain-of-function mutation, E50K acts also via similar mechanisms as the wild type overexpression to more dramatically impair the Tf trafficking. A growing number of mutations in trafficking proteins have been linked with neurodegenerative diseases [46], [47] and glaucoma is also recognized as a neurodegenerative disorder. Based on our current and previous [25] findings, we surmise that the defective trafficking, along with fragmentation of the Golgi complex [48] and increased apoptosis [49], [50], may be the underlying basis how the E50K optineurin mutation renders the patients predisposed to the glaucoma pathology. Correction or restoration to the normal trafficking process by TfR may be a strategy that merits exploration and further investigation.

It is further of note that while the E50K finding has pathologic significance, the wild type optineurin overexpression results are also of physiologic relevance. Optineurin, for example, has been shown to be upregulated by proinflammatory cytokines tumor necrosis factor-α (TNF-α) [13], [51] and interferon [13]. Its expression therefore may be heightened to set off adverse consequences upon chronic inflammation and infection. Increases of TNF-α in the retina and the optic nerve head have also been associated with glaucomatous conditions [52].

## Materials and Methods

### Cell Cultures and transfection

The RPE cell line ARPE-19 obtained from American Type Culture Collection (Masnassas, VA) and RGC5 cells [32] were grown in complete medium containing Dulbecco's modified Eagle's medium (DMEM; Invitrogen, Carlsbad, CA), 10% fetal bovine serum, essential and nonessential amino acids, and antibiotics. Normal human TM tissues excised from eyes of donors (Illinois Eye Bank, Chicago, IL) 24, 38, and 47 years of age were cultured on Falcon Primaria flasks in complete medium [25], [35]. When cells reached confluence, they were trypsinized and subcultured.

Transient transfection was carried out using FuGENE6 (for RPE and TM cells, Roche, Indianapolis, IN) or lipofectamine LTX and Plus reagent (for RGC5 cells, Invitrogen) as previously described [25], [32], [36].

Tet-on OPTN_WT_-GFP inducible stable RGC5 cell line was established as previously described [35]. Tet-on inducible OPTN_E50K_-GFP and OPTN_L157A_-GFP RGC5 cell lines were in addition created following the same procedures and strategies. The only exception was that the OPTN_WT_-GFP fragment was replaced with OPTN_E50K_-GFP or OPTN_L157A_-GFP fragment during the first cloning step [36]. The cells were maintained in DMEM complete medium with 10% Tet system certified fetal bovine serum (Invitrogen), essential and nonessential amino acids, and antibiotics. To induce expression of OPTN_WT_-GFP, OPTN_E50K_-GFP, and OPTN_L157A_-GFP, cells were treated for 16 h with doxycycline (1 µg/ml) (Clontech, Mountain View, CA) in DMEM complete medium.

### Plasmid construction

Plasmids pOPTN_WT_-GFP, pOPTN_E50K_-GFP, pTarget-FLAG-OPTN_WT_, pTarget-FLAG-OPTN_E50K_, pMyocilin-GFP, pRab8_Q67L_-EGFP, and pTfR-EGFP were constructed as previously described [25], [32], [35], [36]. Human myosin VI full length fused to EGFP (pMyoVI-EGFP) was generously provided by Dr. Tama Hasson, University of California San Diego. Plasmid pOPTN_L157A_-GFP was generated by QuikChange II Site-Directed Mutagenesis Kit (Stratagene, La Jolla, CA). The sense primer sequence used for mutagenesis was 5′-CAGAGAAGGCAGACGCGTTGGGCATCGTGTC-3′. The antisense primer sequence was the reverse. To generate open reading frame of Rab5, Rab7, Rab8 and Rab11, cDNAs were synthesized from total RNA of RPE cells by Superscript II cDNA synthesis kit (Invitrogen). Rab4 cDNA was obtained from University of Missouri-Rolla cDNA resource center (Rolla, MO). The cDNAs were used as templates for PCR amplification with primers: 5′-GGCGAATTCTATGTCGCAGACGGCCATGTC-3′ and 5′-GGCGGATCCCTAACAACCACACTCCTGAGCG-3′ for Rab4; 5′- GGCGAATTCTATGGCTAGTCGAGGCGCAAC-3′ and 5′-GGCGGATCCTTAGTTACTACAACACTGATTCCTGG-3′ for Rab5; 5′- GGCGAATTCTATGACCTCTAGGAAGAAAGTG-3′ and 5′-GGCGGATCCTCAGCAACTGCAGCTTTCTG-3′ for Rab7; 5′- GGCGAATTCTATGGCGAAGACCTACGATTA-3′ and 5′- GGCGGATCCTCACAGAAGAACACATCGGA-3′ for Rab8; 5′- GGCGAATTCTATGGGCACCCGCGACGACGA-3′ and 5′- GGCGGATCCTTAGATGTTCTGACAGCACTGCACC-3′ for Rab11. The resulting PCR products were digested with *EcoR*I and *BamH*I and cloned in frame into pEGFP-C1 (BD Biosciences, San Jose, CA) at the same restriction sites to yield constructs pGFP-Rab4, -Rab5, -Rab7, -Rab8 and -Rab11. Plasmids pOPTN_WT_-His, pOPTN_E50K_-His and pOPTN_L157A_-His were made by adding a His tag to the C-terminal of optineurin by PCR using pOPTN_WT_-GFP, pOPTN_E50K_-GFP or pOPTN_L157A_-GFP as the template and the subsequent subcloning of OPTN_WT_-His, OPTN_E50K_-His or OPTN_L157A_-His into EcoR V linearized pcDNA3.1z vector (Clontech). Sequencing was followed to verify all the constructs.

### Antibodies

The following antibodies were used: rabbit polyclonal anti-optineurin (Cayman Chemical, Ann Arbor, MI), anti-GFP (Santa Cruz Biotechnology, Santa Cruz, CA), anti-GAPDH (Trevigen, Gaithersburg, MD), anti-His (Santa Cruz), and anti-β1-integrin (GIBCO-BRL, Grand Island, NY), and mouse monoclonal anti-human TfR (Zymed Laboratories, San Francisco, CA), anti-FLAG (Sigma, St. Louis, MO), anti-EEA1 (BD Biosciences), anti-myosin VI (sigma), anti-Rab8 (BD Biosciences), anti-His (Sigma) and anti-GFP (BD Biosciences).

### Tf uptake and quantification

Cells grown on glass chamber slides were washed in phosphate-buffered saline (PBS) and incubated in DMEM containing 0.2% bovine serum albumin (BSA) (DMEM-BSA) for 1 h to deplete serum. The cells were transferred to fresh DMEM-BSA containing 25 µg/ml TR-Tf (Invitrogen) and incubated for the indicated time periods. The cells were then placed on ice, washed with cold PBS containing 0.2% BSA, 1 mM CaCl_2_ and 1 mM MgCl_2_, incubated with cold acid buffer (0.2 M acetic acid, 0.5 M NaCl) and ice-cold PBS, fixed, and mounted in Vectashield with DAPI (Vector Laboratories, Burlingame, CA). Photography was carried out on a Leica SP2 confocal system (Leica Microsystems, Bannockburn, IL) with a 63× oil objective using sequential scanning to minimize the bleed through. Confocal images were captured using the same resolution, zoom, pinhole size, and amplitude offset. All cells were captured at the same gain. TR-Tf internalization at each time point was quantified as previously described [53]. In brief, using the Leica confocal software, the average grayscale pixel intensity was measured in a small region without any staining. This value was defined as background and the threshold of the red channel was set to this value. Since the transfection efficiency of plasmid construct was relatively low in contrast to high efficiency of siRNA transfection (>95%), two different methods were applied to measure internalized TR-Tf intensity. For cells transfected with GFP constructs, the outline of single cells was drawn and the fluorescence intensity was measured from inside of the cell. The resulting red intensity at each time point was averaged from cells in seven 20× fields in which at least 8 untransfected and 8 transfected cells were selected. For siRNA transfected cells, seven 20× fields, each containing 30–50 transfected cells, were chosen. The total red intensity was acquired and normalized to the number of cells in each field. Three sets of independent experiments were carried out. The significance of the data was determined by Students' t tests.

### Immunoblotting

Cells were washed with ice-cold PBS and harvested in CelLytic^TM^-M cell lysis reagent (Sigma) containing protease inhibitor cocktail (Complete; Roche, Indianapolis, IN). The lysates were centrifuged at 12,000× g for 10 min at 4°C. Protein concentration in lysates was determined using the bicinchoninic acid (BCA) assay (Pierce, Rockford, IL) per the manufacturer's protocol. Aliquots of total cell lysate were separated on sodium dodecyl sulfate-polyacrylamide gel electrophoresis (SDS-PAGE) and electroblotted onto Protran BA83 nitrocellulose membrane (Whatman, Sanford, ME). After blocking for 1 h with 5% nonfat dry milk in a buffer containing 20 mM Tris buffer (pH 7.4), 150 mM NaCl and 0.1% Tween 20, the membrane was incubated with primary antibody for 1 h at room temperature or overnight at 4°C. The blot was further incubated for 1 h with horseradish peroxidase-conjugated secondary antibody (1∶10,000; Jackson ImmunoResearch Laboratories, West Grove, PA). Protein bands were detected using SuperSignal Substrate (Pierce). For repeated probing, the blot was stripped for 1 h at room temperature with ImmunoPure IgG Elution buffer (Pierce).

### Immunofluorescence microscopy

Cells (18,000 cells/well) on Lab-Tek 8-well CC2 glass chamber slides (Nalge Nunc, Rochester, NY) were transfected and fixed with 4% paraformaldehyde in PBS for 15 min. After permeabilization in 0.2% Triton X-100 for 5 min, the cells were blocked with 3% BSA for 30 min, and incubated with primary antibody in blocking solution for 1 h at room temperature. They were further incubated for 45 min with Cy3- or FITC-conjugated secondary antibody (1∶200; Jackson ImmunoResearch Laboratories), and mounted. Confocal images were captured.

### Co-IP using transient transfection or inducible cell lines

Cells plated on 6-well plates were transfected with indicated plasmids for 16 h. For inducible cell lines, the cells were treated with 1 µg/ml of doxycycline for 16 h. After washes with ice-cold PBS, the cells were harvested in IP buffer (50 mM Tris-HCl, pH 7.5, 150 mM NaCl, 1% Nonidet P40, 0.5% sodium deoxycholate, protease inhibitor cocktail) and homogenized by ten strokes in a pre-chilled dounce homogenizer on ice. The homogenates were clarified by centrifugation at 12,000× g for 10 min at 4°C. Protein concentration in homogenates was determined by BCA assay. Aliquots of the homogenates were subjected to immunoblotting or IP reaction using Catch and Release v2.0 kit (Millipore, Billerica, MA) according to manufacturer's protocol with minor modifications. Briefly, 500 µg of the homogenates were incubated overnight with 4 µg of indicated rabbit polyclonal antibodies and 10 µl of Antibody Capture Affinity Ligand (Millipore) on a rotary shaker with gentle shaking at 4°C. The mixture was transferred to a spin column containing agarose resin slurry and further incubated for 30 min on a rotary shaker at room temperature. Columns were washed three times with 1× wash buffer (Millipore). Protein was eluted with 1× denaturing elution buffer (Millipore). The eluates were subjected to SDS-PAGE and immunoblotting.

### siRNA-mediated knock down of optineurin

RPE cells were plated in complete media at 80 to 90% confluency overnight. The cells were transfected with 50 nM of siRNA duplex (Dharmacon, Lafayette, CO) for 2 days using TransIT-TKO transfection reagent (Mirus Bio Corporation, Madison, WI) according to the manufacturer's protocol. Target sequence of optineurin siRNA was 5′-GAAGCCATGAAGCTAAATA-3′ and matched to 166–184 bp of ORF. As a control, a 19-nucleotide scrambled generic control siRNA (Dharmacon) was used. In some experiments, cells were transfected with scrambled or optineurin siRNA along with Cy3-labeled control siRNA (Dharmacon) to assess transfection efficiency.

### Optineurin foci counting

RPE and TM cells were transfected with pEGFP-N1, pOPTN_WT_-GFP or pOPTN_L157A_-GFP for 16 h. Images were taken using a 63× oil objective. The number of foci formed was counted in at least 60 transfected cells both manually and through the use of Metamorph software. The significance of the data was determined by Students' t tests. Three sets of independent experiments were analyzed.

### Biotinylation of cell surface proteins

Surface biotinylation was performed as described [53], [54] with modifications. Briefly, RPE cells after transfection for 16 h were washed with ice-cold PBS. They were then incubated for 20 min at 4°C in a 1 ml solution containing the non-permeable Sulfo-NHS-SS-Biotin reagent (1 mg/ml in PBS). The cells were washed with PBS plus 100 mM lysine for 20 min to quench the reagent. After additional washes, the cells were lysed with lysis buffer for 30 min. The biotinylated proteins were recovered by incubating the lysate for 2 h at room temperature with streptavidin–agarose beads. After washes of the beads, the protein bound to the beads was separated by SDS/PAGE and immunoblotted with anti-TfR for the biotinylated surface TfR. As a control, the biotinylated β1-integrin, a cell surface protein, was probed with anti-β1-integrin.
